# Can We Noninvasively Rule Out Acute Rejection? External Validation of a Urinary Chemokine-Based Model

**DOI:** 10.3389/ti.2024.13810

**Published:** 2024-12-04

**Authors:** Ilaria Gandolfini, Benedetta Mordà, Elena Martinelli, Marco Delsante, Giovanni Maria Rossi, Micaela Gentile, Sara Alibrandi, Daniel Salvetti, Omar Ben Youssif, Enrico Fiaccadori, Alessandra Palmisano, Paolo Cravedi, Umberto Maggiore

**Affiliations:** ^1^ Nephrology Unit, University Hospital of Parma, Parma, Italy; ^2^ Department of Medicine and Surgery, University of Parma, Parma, Italy; ^3^ Translational Transplant Research Center and Department of Medicine, Icahn School of Medicine at Mount Sinai, New York, NY, United States

**Keywords:** biomarkers, chemokine CXCL9, chemokine CXCL10, graft rejection, kidney transplantation (KT)

Dear Editors,

One of the major unmet needs of kidney transplantation is the availability of validated biomarkers for the noninvasive diagnosis of rejection [1]. This is especially true in clinically stable patients at low immunological risk [2], who are less likely to benefit from invasive surveillance biopsies. Emerging evidence support the combined use of noninvasive biomarkers and clinical parameters for risk-stratification [3–5].

A large multicentric cohort study showed that adding plasma donor-derived cell-free DNA (dd-cfDNA) to a standard of care prediction model improves discrimination for acute rejection in kidney transplant recipients (KTRs) [4]. However, dd-cfDNA is less sensitive in detecting T-cell-mediated rejection (TCMR) compared to antibody-mediated rejection (ABMR), especially when early and borderline lesions are present [6, 7].

Therefore, interest in alternative biomarkers of TCMR, including urinary chemokines CXCL9 and CXCL10, is growing [5, 8, 9]. Thanks to the availability of the Ella Automated Immunoassay System, multiple urinary chemokines can be inexpensively quantified in urine supernatant [3]. Recently, a large single-center prospective cohort study developed a predictive model for acute rejection (AR) based on integrating urinary chemokines with routine clinical markers, such as BK Polyoma virus (BKPyV) DNAemia, presence of circulating donor-specific anti-HLA antibodies (DSAs), and eGFR (MDRD formula). The model has a high diagnostic discriminatory value for detecting AR (ROC AUC 81.3%) [3]. The authors argued that implementing this model would allow avoiding 59% of the biopsies, as patients classified at low AR risk could safely skip the biopsy [3]. One potential limitation of this model is the fact that BKPyV DNAemia and urinary chemokine measurements may suffer from large inter-laboratory variability. Therefore, the predictive performance of the model might deteriorate upon validation in external and completely independent cohorts that use different labs.

Herein, we aimed to externally validate the model in a consecutive series of KTRs who underwent a for-cause or surveillance kidney biopsy at the University Hospital of Parma, Parma, Italy. The study was approved by the local Institutional Review Board (IRB) (Protocol #46898, 24/11/2020), and all the patients signed informed consent to the study.

Mid-stream urinary samples were collected on the day of the biopsy (before the procedure) for urinary chemokine analyses. The samples were centrifuged, and the urine supernatants were frozen at −80°C within 4 h from the collection, as previously described [8]. Thawed samples were run in batches on Simple Plex assay for dual detection kit for CXCL9 and CXCL10 (Biotechne, Minnesota, USA. cat# SPCKC-PS-001623). For the analyses, we considered the average of the triplicate values. BKPyV DNAemia copies were detected using real-time PCR and DSAs were detected by Luminex xMAP (LIFEcodes Class I and II kit, Immunocor).

We included 124 kidney transplant recipients (N = 21 with AR), aged 48.5 ± 12.7 years. As shown in Supplementary Table S1, 62.1% were males, 10.5% received a living donation, 12.9% were re-transplantation, and 3 patients (2.4%) received ABO/HLA incompatible kidneys. The patients with a diagnosis of AR received more often Thymoglobulin induction (35.0% vs. 13.6%, P = 0.045). Acute rejection episodes were T-cell mediated in 10 (47.6%) of the cases and antibody mediated or mixed in the remaining ones. At the time of biopsy, DSAs were detected more often in the rejecting patients (28.6% vs. 6.8%, P = 0.009), while there was no difference in MDRD eGFR at the biopsy and in BKPyV DNAemia positivity or copies/mL (Supplementary Table S1). The diagnostic performance of urinary chemokines in this cohort is reported in the Supplementary Material. Figure 1 shows the calibration plot of observed against expected probabilities of AR [10]: calibration is plotted in groups across the AR risk spectrum, and via a smoothed regression line, both with the associated 95% confidence intervals (see Supplementary Material, for further details).

**FIGURE 1:**
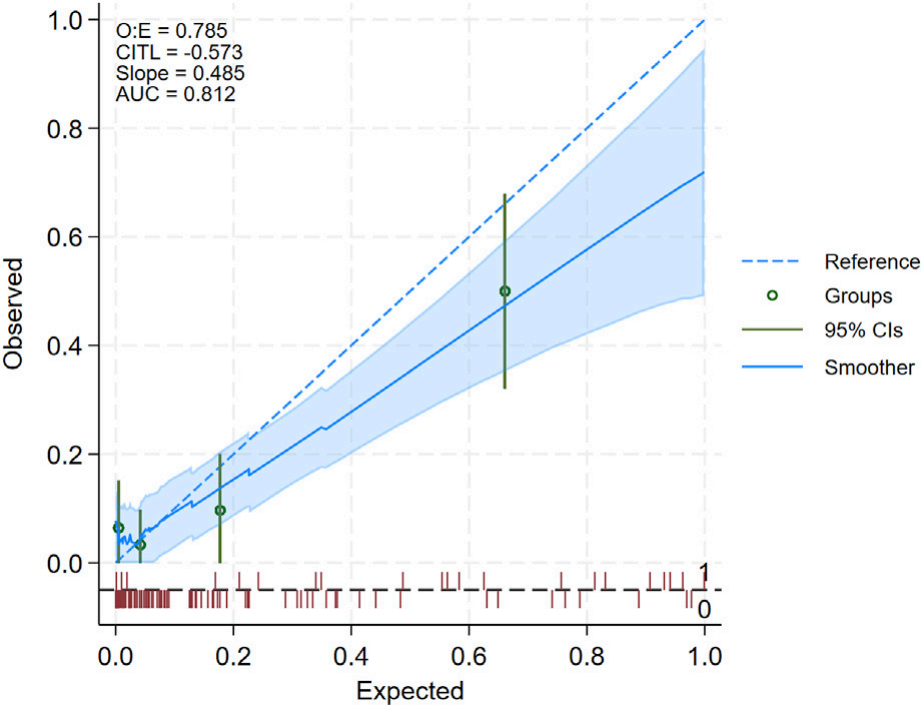
Calibration plot summarizing the results of model external validation. The diagonal dotted blue line represents the line of identity between observed and expected acute rejection (AR) positive biopsies, while the solid blue line represents the smoothed regression line: a perfect model prediction would cause the solid blue and dotted blue line to overlay exactly. When the solid blue line is above the dotted blue lines, the model underestimates the AR risk, if it is below, it overestimates the risk. The shaded area represents the 95% confidence interval of the regression line: if the dotted line falls within the margin of the shaded area, then the difference between the observed and predicted can be regarded as statistically non-significant. Another hint to infer whether the difference is statistically significant is based on the green dots and the green vertical lines representing, for each quartile of AR risk, the estimated observed risk and 95% confidence interval: if the vertical green line does not cross the dotted blue line, then the difference between observed and expected can be regarded as statistically non-significant. The red rug (spike) plot at the bottom represents the number of patients, with positive (=1, above the dotted gray horizontal line) and negative (=0, below the gray horizontal line) biopsies. In the upper left corner are reported the ratio of observed to expected positive biopsies (O:E), Calibration-In-The-Large (CITL) namely, the average predicted AR risk is compared with the overall event rate, the slope of the regression line of observed vs. expected, and the Area Under (AUC) of the ROC curve.

The plot shows that the model’s expected and observed AR risks align in patients at the lower risk end of the spectrum. Consistently, the shaded blue area, which represents the 95% confidence interval of the regression line, and the 95% confidence interval of the quartile of AR risk (vertical green line), included the line of identity for the lower bounds of AR risk (left-hand side of the plot). In contrast, for expected AR risk above approximately 0.4 (i.e., 40%, right-hand side of the plot), the model tended to overestimate the risk of AR. The upper left corner of the plot reports the performance statistics which confirmed that predicted AR risk slightly overestimated observed AR risk, as the value of the observed to expected ratio (O:E) and of the slope were both below 1, and the value of the CITL (Calibration-In-The-Large, i.e., average predicted AR risk is compared with the overall event rate) was below zero. On the other hand, the AUC of the ROC curve (81.2%) showed good model discrimination.

We acknowledge that model validation was carried out on a limited number of subjects compared to the original cohort. However, this is, to the best of our knowledge, the first attempt to validate an integrated model based on urinary chemokines CXCL9 and CXCL10 in an independent cohort of subjects. Moreover, our findings are remarkably similar to those of the original cohort. In fact, discriminatory capacity was identical to that estimated in the original cohorts (AUC of the ROC curve 81.2% [95 percent confidence interval: 69.1 to 93.2] vs. 81.3% of the original study). The model on average, overestimates the risk of AR, a trend which was also partially observed in the original study [3]. However, overestimation occurred only for patients at the higher AR risk of the spectrum. We also drew a Decision Curve Analysis (Supplementary Figure S1), which confirmed that the model is useful for decision-making purposes for threshold probabilities up to 50% (the threshold probability is the minimum probability of AR at which a decision-maker would take the decision to perform a biopsy).

In conclusion, our findings on an independent cohort of patients support the utility of this model for identifying patients at low risk of AR in whom biopsy can be safely avoided.

## Data Availability

Datset will be made available to other researchers following publication upon request. Further inquiries can be directed to the corresponsing author.
