# Supplementary material for: Can We Noninvasively Rule Out Acute Rejection? External Validation of a Urinary Chemokine-Based Model
Source: Transpl Int. 2024 Dec 4;37:13810. doi: 10.3389/ti.2024.13810 (PMC11652145; doi:10.3389/ti.2024.13810)
Supplement: Supplementary file 1 [file DataSheet1.docx]

**Supplementary appendix**

**Statistical methods for model validation**

We used calibration plots to summarize the results of model external validation [1]. Calibration plot is a visual tool to assess the agreement between predictions and observations across the different levels of the predicted probability. The diagonal dotted blue line represents the line of identity between observed and expected acute rejection positive biopsies, while the solid blue line represents the smoothed regression line: a perfect model prediction would cause the solid blue and dotted blue line to overlay exactly. When the solid blue line is above the dotted blue lines, the model underestimates the acute rejection risk, if it is below, it overestimates the risk. The shaded area represents the 95% confidence interval of the regression line: if the dotted line falls within the margin of the shaded area, then the difference between the observed and predicted can be regarded as statistically non-significant. Another hint to infer whether the difference is statistically significant is based on the green dots and the green vertical lines representing, for each quartile of acute rejection risk, the estimated observed risk and 95% confidence interval: if the vertical green line does not cross the dotted blue line, then the difference between observed and expected can be regarded as statistically non-significant. The red rug (spike) plot at the bottom represents the number of patients, with positive (=1, above the dotted gray horizontal line) and negative (=0, below the gray horizontal line) biopsies. We additionally calculated numerical statistics expression the calibration namely, the ratio of observed to expected positive biopsies (O:E), Calibration-In-The-Large (CITL), the average predicted acute rejection risk compared with the overall event rate, the slope of the regression line of observed vs expected in the calibration plot [1]. We used the receiver operating characteristic area under the curve (ROC AUC) to evaluate discrimination. We calculated the 95% confidence interval of the ROC curve using the method of DeLong, DeLong, and Clarke-Pearson [2]. We employed decision curve analysis (DCA) to examine the added value of the predictive model including the urinary chemokines for clinical decision making [3, 4]. DCA enables appraising the added value of a diagnostic tool, namely the acute rejection predictive model, after considering the model false positive and false negative rates. DCA is carried out as follows [3, 4]: a reasonable range of threshold probability of acute rejection to perform a biopsy is chosen and plotted on x-axis. The net benefit of the biopsy is plotted on the y-axis. Net benefit, which is defined as benefit − (harm × threshold probability), is calculated according to the following equation: TPR − FPR × t, with TPR being true positive rate (acute rejection), FPR being false rate (both calculated with respect to the total sample size), and t the probability threshold expressed as odds. Physicians, considering patient preferences, may vary in their propensity to perform a biopsy. One can then consider the benefit/harm ratio in performing a biopsy across several individual scenarios. Each individual scenario is given by the combination of the chosen threshold probability and the chosen strategy. The threshold probability is the minimum probability of acute rejection at which a decision-maker would take the decision to perform a biopsy. The strategy is the use or the non-use of the predictive model to take the decision of performing the biopsy. The DCA is represented by a plot that reports three lines on the relation between Net benefit and threshold probability, as follows: the line representing the strategy of performing a biopsy on all patients (blue); the line representing the strategy of using the model for deciding upon who should be biopsied (green); the line representing the strategy of no biopsy whatever the probability (red). In the DCA plot, for each given threshold probability (x-axis), the higher the line the greater is the Net benefit.

[1] Steyerberg EW, Vergouwe Y. Towards better clinical prediction models: seven steps for development and an ABCD for validation. Eur Heart J. 2014;35:1925-31. doi: 10.1093/eurheartj/ehu207.

[2] DeLong, E. R., D. M. DeLong, and D. L. Clarke-Pearson. 1988. Comparing the areas under two or more correlated receiver operating characteristic curves: A nonparametric approach. Biometrics 1988; 44: 837-845.

[3] Vickers AJ, van Calster B, Steyerberg EW. Net benefit approaches to the evaluation of prediction models, molecular markers, and diagnostic tests. BMJ. 2016;352:i6. doi:10.1136/bmj.i6

[4] Vickers AJ, van Calster B, Steyerberg EW. A simple, step-bystep guide to interpreting decision curve analysis. Diagn Progn Res. 2019;3:18. doi:10.1186/s41512-019-0064-7

**Analysis on urinary chemokines performance in the current validation cohort**. In the current cohort, the empirical optimal cutpoint (calculated with the Liu method that maximizes the product of the Sensitivity and Specificity [1]) for log – CXCL9 to Creatinine ratio, mg/mmol was 2.716 (Sensitivity at cutpoint: 86%; Specificity at cutpoint: 71%); the empirical optimal cutpoint for log – CXCL10 to Creatinine ratio, mg/mmol was 1.334 (Sensitivity at cutpoint: 95%; Specificity at cutpoint: 68%).

Integrated Discrimination Improvement (i.e., the proportion of improved correct classification provided by the new biomarker [2]) were as follows: for log – CXCL9 to Creatinine ratio compared to the use of eGFR, DSA, and BKPyV DNA only, was 0.1360 (one-sided P value= 0.0404); for log – CXCL9 to Creatinine ratio it was 0.1474 (one sided P value = 0.0445). In other words, each individual biomarker improved the percentage of correctly classified by 14% with respect to the use of eGFR, DSA and BKPyV DNA only.

[1] Liu X. Classification accuracy and cut point selection. Stat Med. 2012;31(23):2676-86. doi: 10.1002/sim.4509

[2] Pencina MJ, D'Agostino Sr RB, D'Agostino Jr RB, Vasan RS. Evaluating the added predictive ability of a new marker: From area under the ROC curve to reclassification and beyond. Stat Med. 2008; 27:157-172.

| **Table S1. Population characteristic by rejection on biopsy** | | | | |
| --- | --- | --- | --- | --- |
|  | **No rejection**  **(N=103)** | **Rejection**  **(N=21)** | **Total**  **(N=124)** | **P value** |
| Age at transplant, years | 48.7 ± 13.3 | 47.4 ± 10.0 | 48.5 ± 12.7 | 0.503 |
| Male sex | 63 (61.2%) | 14 (66.7%) | 77 (62.1%) | 0.806 |
| Primary kidney disease |  |  |  |  |
| *Glomerulonephritis* | 20 (19.4%) | 3 (14.3%) | 23 (18.5%) | 0.291 |
| *CM-TMA/C3GN* | 5 (4.9%) | 0 (0.0%) | 5 (4.0%) |  |
| *Diabetes* | 3 (2.9%) | 1 (4.8%) | 4 (3.2%) |  |
| *ADPKD* | 10 (9.7%) | 4 (19.0%) | 14 (11.3%) |  |
| *CAKUT* | 13 (12.6%) | 0 (0.0%) | 13 (10.5%) |  |
| *Others/Unknown* | 52 (50.5%) | 13 (61.9%) | 65 (52.4%) |  |
| Hypertension | 74 (71.8%) | 16 (76.2%) | 90 (72.6%) | 0.793 |
| Diabetes | 7 (6.8%) | 5 (23.8%) | 12 (9.7%) | 0.031 |
| Donor type (living) | 13 (12.6%) | 0 (0.0%) | 13 (10.5%) | 0.122 |
| Donor's age, years | 53.0 ± 14.5 | 53.2 ± 16.3 | 53.0 ± 14.7 | 0.890 |
| Cerebrovascular death (deceased donor) | 38 (36.9%) | 6 (28.6%) | 44 (35.5%) | 0.618 |
| ABO/HLA-incompatible | 2 (1.9%) | 1 (4.8%) | 3 (2.4%) | 0.430 |
| Re-transplantation | 12 (11.7%) | 4 (19.0%) | 16 (12.9%) | 0.472 |
| Delayed graft function | 23 (22.5%) | 6 (30.0%) | 29 (23.8%) | 0.566 |
| Thymoglobulin induction | 14 (13.6%) | 7 (35.0%) | 21 (17.1%) | 0.045 |
| Basiliximab induction | 88 (85.4%) | 12 (60.0%) | 100 (81.3%) | 0.013 |
| Maintenance |  |  |  |  |
| *Cyclosporine* | 2 (1.9%) | 1 (4.8%) | 3 (2.4%) | 0.430 |
| *Tacrolimus* | 101 (98.1%) | 20 (95.2%) | 121 (97.6%) |  |
| *Steroids* | 103 (100.0%) | 21 (100.0%) | 124 (100.0%) | . |
| *Mycophenolate/Azathioprine* | 103 (100.0%) | 21 (100.0%) | 124 (100.0%) | . |
| *mTOR-inhibitors* | 3 (2.9%) | 0 (0.0%) | 3 (2.4%) | 1.000 |
| eGFR-MDRD at biopsy, ml/min/1.73m^2^ | 48.1 ± 20.1 | 41.0 ± 23.0 | 46.9 ± 20.7 | 0.153 |
| log - CXCL10 to Creatinine ratio, mg/mmol | 0.9 (0.4-1.5) | 2.3 (1.7-2.7) | 1.1 (0.5-1.9) | <0.001 |
| log - CXCL9 to Creatinine ratio, ng/mmol | 2.3 (1.3-3.0) | 3.7 (3.0-4.0) | 2.4 (1.5-3.5) | <0.001 |
| Tacrolimus blood levels, ng/mL | 7.8 ± 5.1 | 6.0 ± 3.4 | 7.5 ± 4.9 | 0.306 |
| Urinary albumin-to-creatinine ratio, mg/gr | 49.0  (16.3-193.3) | 91.0  (21.3-346.6) | 54.1  (17.7-212.3) | 0.309 |
| Urinary protein-to-creatinine ratio, gr/gr | 0.2 (0.1-0.4) | 0.5 (0.2-0.9) | 0.2 (0.1-0.5) | 0.154 |
| Positive plasma BKPyV | 12 (11.7%) | 2 (9.5%) | 14 (11.3%) | 1.000 |
| Plasma BKPyV DNA, copies/mL | 2911 (500-34485) | 3725 (500-22779) | 2911 (500-34485) | 0.591 |
| Anti-HLA antibodies | 27 (26.7%) | 7 (35.0%) | 34 (28.1%) | 0.586 |
| Donor-specific anti-HLA antibodies | 7 (6.8%) | 6 (28.6%) | 13 (10.5%) | 0.009 |
| Protocol Biopsy | 46 (44.7%) | 6 (28.6%) | 52 (41.9%) | 0.227 |
| Borderline rejection | 13 (12.6%) | 0 (0.0%) | 13 (10.5%) | 0.122 |
| TCMR | 0 (0.0%) | 10 (47.6%) | 10 (8.1%) | <0.001 |
| Early/late active ABMR | 0 (0.0%) | 11 (52.4%) | 11 (8.9%) | <0.001 |
| Transplant glomerulopathy | 1 (1.0%) | 0 (0.0%) | 1 (0.8%) | 1.000 |
| BKPyVAN | 2 (1.9%) | 0 (0.0%) | 2 (1.6%) | 1.000 |
| GN recurrence | 1 (1.0%) | 0 (0.0%) | 1 (0.8%) | 1.000 |
| Any lesions on biopsy | 21 (20.4%) | 21 (100.0%) | 42 (33.9%) | <0.001 |
| Active cellular inflammatory injury | 15 (14.6%) | 10 (47.6%) | 25 (20.2%) | 0.002 |
| Mann-Whitney test for continuous variables (reported as mean ± standard deviation or median (min - max)). Fisher's exact test for categorical variables (reported as number (percentage)). Baseline characteristics of the study population ABMR antibody mediated rejection; ABO/HLA-incompatible ABO blood group or Human Leukocyte Antigen incompatible, ADPKD, Autosomal dominant polycystic kidney disease; BKPyV, BK polyoma virus; BKPyVAN, BK polyoma virus nephropathy; CAKUT, congenital anomalies of the kidneys and urinary tracts; CM-TMA/C3GN, complement-mediated thrombotic microangiopathy/C3 glomerulonephritis; CMV-R+, cytomegalovirus positive recipient; CNI, calcineurin inhibitors; eGFR, estimated GFR (MDRD); GN, glomerulonephritis; OPTN-KDPI, organ procurement and transplantation network kidney donor profile index; sCreatinine, serum Creatinine; TCMR T-cell mediated rejection. | | | | |

**Figure S1**

**
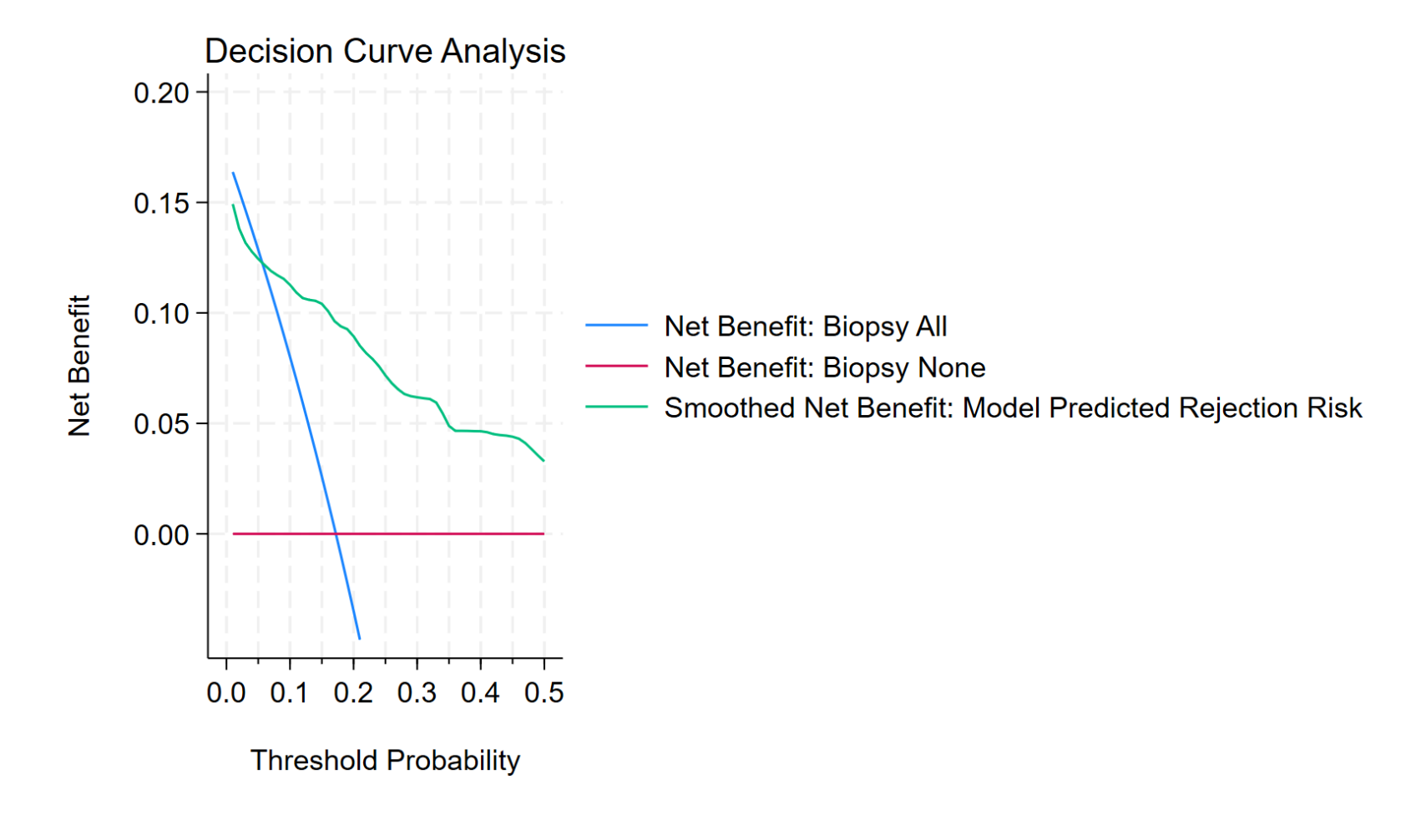
**

**Figure S1.** Decision curve analysis: plot of the Net Benefit (difference between true positives and false positives times the odds of threshold probability) and the patient risk of acute rejection (AR). The threshold probability is minimum probability of AR at which a decision-maker would take the decision to perform a biopsy. When the green line is above the blue line, the strategy of using the model for deciding upon who should be biopsied provides more benefit compared to a strategy of doing biopsy to all patients; if the green line falls below the red line, then the strategy of using the model for such a decision is no longer useful.
